# Characterization of Highly Filled Glass Fiber/Carbon Fiber Polyurethane Composites with the Addition of Bio-Polyol Obtained through Biomass Liquefaction

**DOI:** 10.3390/ma14061391

**Published:** 2021-03-12

**Authors:** Adam Olszewski, Paweł Nowak, Paulina Kosmela, Łukasz Piszczyk

**Affiliations:** Department of Polymer Technology, Chemical Faculty, Gdansk University of Technology, 80-233 Gdansk, Poland; Adam.Olszewski@pg.edu.pl (A.O.); Pawel.Nowak@pg.edu.pl (P.N.); Paulina.Kosmela@pg.edu.pl (P.K.)

**Keywords:** polyurethane composites, bio-polyol, inorganic fibers, biomass liquefaction

## Abstract

This work aims to investigate the process of obtaining highly filled glass and carbon fiber composites. Composites were manufactured using previously obtained cellulose derived polyol, polymeric methylene diphenyl diisocyanate (pMDI). As a catalyst, dibutyltin dilaurate 95% and Dabco^®^ 33-LV were used. It was found that the addition of carbon and glass fibers into the polymer matrix causes an increase in the mechanical properties such as impact and flexural strength, Young’s modulus, and hardness of the material. Moreover, the dynamic mechanical analysis (DMA) showed a significant increase in the material’s storage modulus and rigidity in a wide range of temperatures. The increase in glass transition of soft segments can be noticed due to the limitation of macromolecules mobility in the material. The thermogravimetric analysis showed a four step decomposition, with maximal degradation rate at TmaxII = 320–330 °C and TmaxIII = 395–405 °C, as well as a significant improvement of thermal stability. Analysis of the material structure using a scanning electron microscope showed the presence of material defects such as voids, fiber pull-outs, and agglomerates of both fibers.

## 1. Introduction

Polyurethane composites are becoming an increasingly popular solution in modern science and industry, due to the increasingly restrictive quality requirements for construction materials, including corrosion resistance, durability, and high strength to weight ratio. These materials have several advantages that make them useful for the production of polyurethane composites. One of the most important advantages is the ability to design the product to meet the established requirements. Polyurethane materials can be produced in various forms, from rigid and crosslinked resins, super lightweight foams, to flexible and durable elastomers. A wide selection of petrochemical and natural intermediates, additives, and fillers allows the manufacturing of materials with specific properties, which include parameters such as tensile and flexural strength, abrasion resistance, apparent density, cost, biodegradability, etc. [1,2].

The most widely used method to enhance the properties of polymeric materials is the addition of organic and inorganic additives into the polymeric matrix. Glass and carbon fibers are among the most popular inorganic fibers used in the production of polymer composites [3], which is due to tremendous chemical stability, lightweight, mechanical properties, and very high temperature resistance. Moreover, these fibers are relatively cheap, which allows the industrial use of these materials. Recently, the addition of inorganic fibers to polyurethane materials has been a frequent topic in scientific research. Recent research showed that hybridization of glass fiber composites can lead to improving the properties of the composites. A replacement of 25% of glass fiber using Zylon/Kevlar fibers may improve the impact performance up to 25% [4]. Moreover, glass fiber composites have decent fatigue behaviour, but the stress concentration may reduce the fatigue life of laminated composites. This effect is magnified with the increasing number of cycles [5].

Scientists in their research are trying to obtain materials with the best available properties from wastes and bio-based products [6,7,8]. Several studies have found that biomass liquefaction is a useful method for obtaining bio-polyols. The increased interest in bio-based raw materials is related to the shrinking oil resources, the constant increase in gas and oil prices, and the implementation of more restrictive policies by the E.U. due to climate change, and the increase in hazardous substances released into the environment.

The available scientific literature widely describes liquefaction as a process of obtaining bio-polyols from various types of biomass, solvents, and catalysts. Mateus et al. [9], in the liquefaction process, used cork powder in a mixture of two solvents—1:1 *w*/*w* of diethylene glycol and 2-ethylhexanol. As a catalyst, 3% of p-toluene sulfonic acid was used. The temperature of the reaction was 160 °C. Sonification at different amplitudes improved biomass conversion. Vale et al. [10] used the same catalyst. In this paper, as a biomass, the author used cork powder and eucalyptus bark. Liquefaction was conducted in a mixture of diethylene glycol and 2-ethyl hexanol in a mass ratio of 1:1:3 (biomass:diethylene glycol:2-ethyl hexanol). Tran et al. [11] used the macroalgae Saccharina japonica as a biomass. To proceed the liquefaction mixture of 20% *w*/*w* PEG 300, poly (ethylene glycol) with molecular weight 300 g/mol and glycerol was used. As a catalyst, 95% sulphuric acid was added to the mixture. In another work, da Silva [12] used three different renewable organic acids (lactic, acetic, and citric) in the liquefaction of pure lignin, as a solvent 80/20% *w*/*w* mixture of PEG 400 and glycerol. The obtained yields were similar, ranging between 85–88%. The liquefied products showed the acid number as less than 10.07 mg KOH/g and hydroxyl groups up to 660.08 mg KOH/g. The molecular weight of the products was in the range of 1459–1990 g/mol^−1^. Zheng et al. [13], in their work, used chitin and shrimp shells by conducting liquefaction in diethylene glycol with the addition of a catalyst—95% sulphuric acid. After this process, the blend of liquefied product with polyvinyl alcohol was used for the production of membranes. Hu et al. [14] conducted the liquefaction of soybean straw (loaded from 10 to 25%) in unrefined crude glycerol. Concentrated 98% sulphuric acid was added as a catalyst (loading from 0 to 5%). The liquefaction was conducted at a preset reaction time (45–360 min) and at a temperature range from 120 to 240 °C. To produce bio-polyols of suitable material properties for polyurethane (PU) production, liquefaction at 240 °C for 180 min, at 3% sulfuric acid loading, and biomass loading of 10–15% was preferred. In the production of PU materials, most research has focused on foamed materials [8,14,15]. However, the area of obtaining cross-linked not foamed polyurethane materials has not been explored in depth, with only a few works on this subject [16,17].

Our previous work [17] has shown the promising properties of biomass-originated polyols and the possibility of manufacturing polyurethanes with the addition of this polyol. Possible applications of the obtained materials are the production of boards, table tops, and laminates. This solution will be a greener alternative to the currently used composites produced from petrochemical raw materials. This work aims to validate using bio-based polyols to manufacture highly filled polyurethane composites and the impact of different kinds of inorganic fibers on their properties. As a reinforcement, up to 50% of carbon and 70% of glass fiber were used. To determine the properties and structure of the obtained composites, a variety of research methods, such as flexural and impact strength, hardness, dynamic mechanical analysis (DMA), thermogravimetry analysis (TGA), Fourier-transform infrared spectroscopy (FT-IR), and scanning electron microscopy (SEM) were used.

## 2. Materials and Methods

### 2.1. Materials

To obtain a bio-based polyol, PEG 400 (polyethylene glycol) supplied by POCH S.A. (Gliwice, Poland) and refined crude glycerol acquired from Bio-Chem sp. z.o.o. (Olszanka, Poland) were used as a liquefaction solvent. The hydroxyl number of bio-based polyol was 475 mg KOH/g and the water content was 0.27%. A concentrated 95% aqueous solution of sulfuric (VI) acid was purchased from Avantor Performance Materials Poland S.A. (Gliwice, Poland) and used as a catalyst. Shredded cellulose (50 μm diameter powder) was obtained from POCH S.A. (Gliwice, Poland). Polyether polyol Rokopol^®^ M6000 (LOH = 28 mg KOH/g) was supplied by PCC Rokita S.A. (Brzeg Dolny, Poland). The polymeric methylene diphenyl diisocyanate (pMDI) was provided by Borsodchem (Kazincbarcika, Hungary). The content of free isocyanate groups in pMDI was 31%. Dibutyltin dilaurate 95% and Dabco^®^ 33-LV were purchased from Sigma-Aldrich (Saint Louis, MO, USA). Glass fiber of 6 mm was purchased from KROSGLASS S.A. (Krosno, Poland) and 6 mm of carbon fiber was purchased from Surfpol (Nowy Kurzeszyn, Poland).

### 2.2. Preparation of Highly Filled Polyurethane Composites

The liquefaction of biomass (cellulose) was carried out in a similar way as in our previous publication [13], in a three-neck reactor under atmospheric pressure. The mixture was stirred using a mechanical stirrer. A heating mantle heated the reactor. In this reaction, a 50/50 wt% mixture of PEG400 and glycerol was used as a solvent and 3 wt% of 95% sulfuric acid was added. The mass ratio of biomass to the solvent was 1:10. In the second step, the obtained product was neutralized with sodium hydroxide (NaOH) to a pH of 7, and then dried under a vacuum for 2 h to remove the excess water. Finally, the hydroxyl number of the polyol after neutralization was determined.

To obtain polyurethane composites, a one-step method using a two-component system was used. Glass or carbon fibers were added to polyol (90% of bio-polyol and 10% of M6000), containing 0.5% by weight of dibutyltin dilaurate and Dabco^®^ 33-LV and stirred in a beaker until a homogeneous mixture (Component A) was obtained. The ratio of the NCO-OH groups was 1:1 (isocyanate index = 1). Components A and B (pure pMDI isocyanate) were mixed. Next, the mixture was poured into the mould and hot pressed for 15 min at 100 °C under a pressure of 10 MPa. After that, the obtained materials were cold pressed for 10 min under a pressure of 10 MPa. The thickness of the samples was 3 mm. The reference bio-based material (Matrix) without a filler, as well as 12 sets of composites with different contents of carbon or glass fiber were fabricated, as shown in Table 1.

### 2.3. Characterization of Polyurethane Composites

The FT-IR spectrophotometric analysis was performed to determine the structure of the obtained polyurethanes. The research was performed at a resolution of 4 cm^−1^ using a Nicolet 8700 apparatus (Thermo Electron Corporation, Waltham, MA, USA) equipped with a Snap-Gold State II, which allows measurements in the reflection configuration mode.

The sample densities were estimated following the standard PN-EN ISO 1183-1:2019-05 [18], using an immersion method for solid plastics. The rectangular samples were measured using an electronic analytical balance with an accuracy of 0.0001 g.

Flexural tests were performed on rectangular specimens following the standard PN-EN ISO 14125:2001 [19]. Samples with normalized dimensions were measured with a slide caliper with an accuracy of 0.1 mm. Tests were performed on a Zwick/Roell 1000 N testing machine at a constant speed of 100 mm/min until fracture. The load was applied in the middle of the freely supported specimen.

The impact strength was performed using the Izod type hammer. The energy of the hammer was 5.5 J. The test was executed according to PN-EN ISO 180:2020-05 [20] and the samples were beam-shaped.

The hardness of the obtained composites was examined using the Shore method. The test was executed according to PN-EN ISO 868:2005 [21].

The dynamic mechanical analysis was performed using the DMA Q800 TA Instruments apparatus. Samples were analyzed in the strain mode with a frequency of 1 Hz. Measurements were performed in the temperature range from −120 to 140 °C with a heating rate of 4 °C/min. Beam-shaped samples with dimensions of 10 mm × 40 mm were used.

To evaluate the thermal stability of materials, the thermogravimetric analysis (TGA) was performed on 10 mg samples using a NETZSCH TG 209 F3 apparatus (NETZSCH, Selb, Germany) under a nitrogen atmosphere in the temperature range from 30 to 800 °C and at a heating rate of 10 °C/min. All specimens were cut out in one piece from the material.

Morphological investigations were performed on the prepared hybrid composites with SEM Quanta FEG 250 (FEI Company, Hillsboro, OR, USA). Before the analysis, the samples were coated with a 10 nm gold layer. The observations were carried out on the samples subjected to brittle cracking in liquid nitrogen.

## 3. Results and Discussion

The mechanical properties and density are essential parameters deciding on the possible future application of the obtained materials. The density, impact strength, and hardness of the samples are shown in Table 2.

The density increases with the higher amount of filler inside the sample, for both carbon and glass fiber filled materials. The density of carbon and glass fibers is higher than the polyurethane bio-based matrix which results in increased values of density for higher filler contents in the material. Due to the relatively low density and good mechanical properties of carbon and glass fibers, the composites have shown a significant improvement in mechanical properties.

Izod impact tests have shown an improvement in the impact strength for all samples in comparison to the matrix. The highest impact strength can be observed for 50 and 60% of filler in glass fiber composites. Carbon fiber composites have shown the highest value for 50% of the filler. However, for 30 and 40% CF the results are high as well, over 2.5 times higher than the value of the matrix impact strength.

Hardness increases with the increase in filler content for both composite types. Materials highly filled with GF (40, 50, and 60% GF) have shown better impact properties than all the carbon fiber filled composites. However, the highest values of flexural strength and Young’s modulus were achieved for the 30% CF composite. It can be concluded that reinforcing with GF could have a higher influence on the impact properties than the similar percentage reinforcing with CF. On the other hand, CF filling provides a higher improvement on Young’s modulus and flexural strength than GF. Carbon fiber composites generally have a slightly higher hardness when using the same level of filling. Densities for both composite types are on a similar level.

Flexural strength, Young’s modulus, and maximal deformation of the composites are presented in Table 3.

Results of flexural tests show that some samples achieved a significant improvement in Young’s modulus and flexural strength compared to the matrix. However, the addition of fibers resulted in the decrease of maximum deformation. Samples containing from 10 to 50% of glass fibers show higher modulus and flexural strength, while the deformation stays on the same level. The sample filled with 60% of glass fibers shows the highest Young’s modulus and flexural strength but the maximal deformation dropped noticeably.

Carbon filled composites, similarly to glass filled, have shown a lower deformation than matrices. All samples showed a significantly higher Young’s modulus than the matrix, with a maximum of almost 3.9 GPa for the sample with 30% filler content. The flexural strength of samples with 10 and 50% of carbon fibers is lower compared to the matrix. The highest values reached samples with 20 and 30% of filler. The best flexural properties show the sample with 30% of filler. Higher contents of fibers were hard to mix effectively due to the increase in viscosity and can contain higher amounts of unreacted compounds and free spaces in the final product, which can drastically decrease the properties of the obtained materials. A high content of filler (especially for the 70% GF) probably resulted in the formation of agglomerates of fibers and, as a result, the presence of irregular voids in the material. These defects caused a significant reduction in the mechanical strength of the material due to the stress concentration, faster formation, and propagation of cracks. Mechanical properties of polyurethane matrix were improved after adding carbon or glass fiber, which is due to the fact that both glass and carbon fibers have very high mechanical properties and stiffness. Composites fibers are taking over the mechanical load, which is applied to the sample. More fibers allow the material to withstand higher loads. However, each defect in the composite leads to a high decrease in the mechanical strength of the composite, and the more filler is present the more often defects occur.

**Figure 1:**
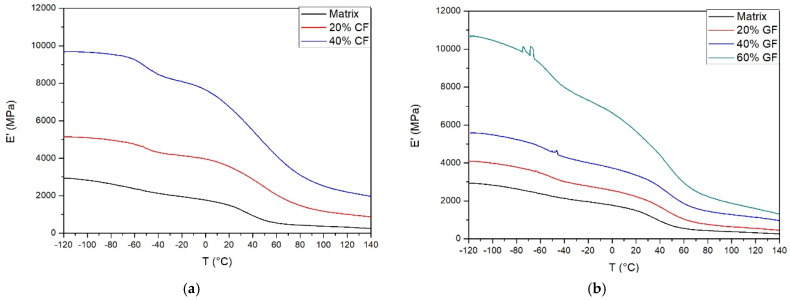
Storage modulus of (**a**) carbon fiber composites, (**b**) glass fiber composites.

The storage modulus provides information on the stiffness, rigidity of composites, and interfaces between phases inside the material. The obtained curves are presented in Figure 1 and the values of E’ at different temperatures are presented in Table 4.

The reinforcing effect from the incorporation of glass and carbon fibers is visible in the increase in the storage modulus compared to the neat polyurethane in a wide range of temperatures, as can be observed. Results show that the value of E’ increases up to 40% CF and 60% GF. At a temperature of 20 °C, this parameter was increased from 1493 MPa for the bio-based matrix to 5667 and 6756 MPa for 60% GF and 40% CF, respectively. The increase in the value of the indicated parameters may be caused by the pinning effect of the carbon and glass fibers in the matrix, therefore inhibiting the polymer chain segment’s movement and enhancing the rigidity of the composites [3,22]. An increase in the storage modulus is also associated with the stress transfer through the matrix material to the glass/carbon fiber. Moreover, it is known that the intermolecular interaction between the polymer structural units and applied fibers affects the storage modulus in a glassy state. The greater the interaction between both phases, the higher E’ is. Analyzing the values given in Table 4 leads to the conclusion that the carbon fiber reinforcement has a higher influence on the mechanical properties than the glass fiber reinforcement. It may be connected to higher mechanical properties of carbon fiber, and to better interactions between the matrix and carbon fiber, compared to the glass fiber. The obtained results are consistent with the performed flexural strength tests [23,24,25]. The greater content of both fibers may cause a decrease in the described modulus, caused by material defects such as voids, agglomerates of the fiber, and previously described material inhomogeneities caused by the manufacturing method.

The increase in temperature causes the transition from the glassy to rubbery state of polymers. The polymer chain segments begin to move with a high internal frictional resistance, causing a rapid drop of the storage modulus. The storage modulus of glass and carbon fiber composites decreases less with the increasing temperature due to the greater resistance of molecular chain motion, which is connected to the mechanical interlocking in the composites [22]. Analyzing the E’ value after the glass transition area, a slight increase in this parameter can be observed, which indicates that there is a significant change in the loss modulus due to the increasing fiber loading and glass fiber ratio at a higher temperature.

**Figure 2:**
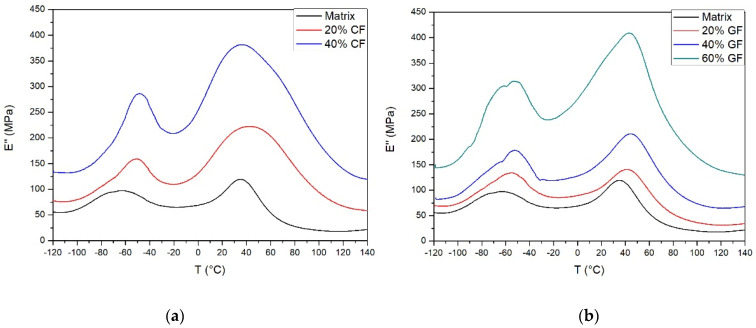
Loss modulus of (**a**) carbon fiber composites, (**b**) glass fiber composites.

The variation of the loss modulus of the obtained materials is presented in Figure 2.

This parameter gives information on the amount of energy dissipated or lost (especially as heat) during the sinusoidal deformation of the sample. The presence of two phases in the structure of the material can be noticed. It can be concluded that both transformations are glass transitions of soft segments. The first peak arrives from the transformation of segments composed of glycerol and condensed glycerol derivates, while the second peak comes from the soft segments composed of the long chain of PEG 400. It can be noticed that the addition of both fibers significantly increased the loss modulus of the composites. Moreover, this parameter improves with the increase in the amount of carbon fibers in the material. In addition, for the material with GF the increase is observed up to the content of 60% GF.

The glass transition temperature of both segments of the obtained materials and the influence of fiber addition on the transition temperatures were analyzed using the loss modulus curves. It was observed that the addition of fibers caused a slight shift of the glass transition temperature of II soft segments towards higher temperatures. This effect may be caused by the restriction of chain mobility connected with the addition of stiff fibers, which was also noticed by other authors. Moreover, a shift in the glass transition temperature of the I soft segments towards higher temperatures was noticed, which also confirms the decrease of the polymer chain mobility in the matrix [26].

**Figure 3:**
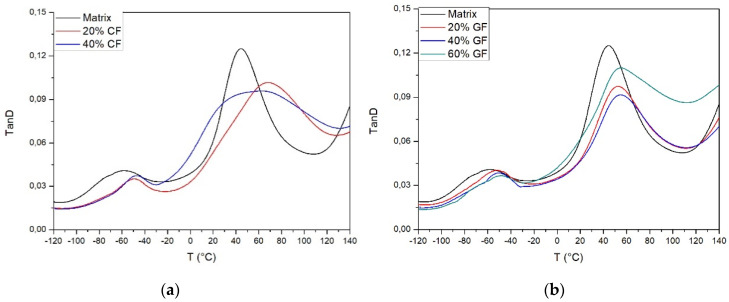
Loss factor (Tan δ) values for (**a**) carbon fiber composites, (**b**) glass fiber composites.

The variation of loss factor for the obtained composites is presented in Figure 3. This parameter is the ratio between both above-mentioned modules and can be correlated with impact toughness. During the analysis, it was noticed that the addition of both fibers caused the decrease of the loss factor. A noticeable drop of the Tan δ value may indicate an increase in the stress transfer efficiency between both phases. Moreover, materials such as glass fibers, may retain energy and avoid energy dissipation. During deformation of the matrix, part of the energy can be retained as a heat, thus it can be considered that the drop of the loss factor may be correlated with the intensified interactions between interfaces at the greater content of both fibers and the effective reinforcement of bio-PU matrix. Nurazzi et. al. [26] in their work have shown that the incorporation of fibers may reduce the value of Tan δ by restriction of the polymer molecules movement. This will increase the damping properties of the manufactured composites. Moreover, it was noticed that the decrease in this modulus may be associated with an increase in impact strength.

**Figure 4:**
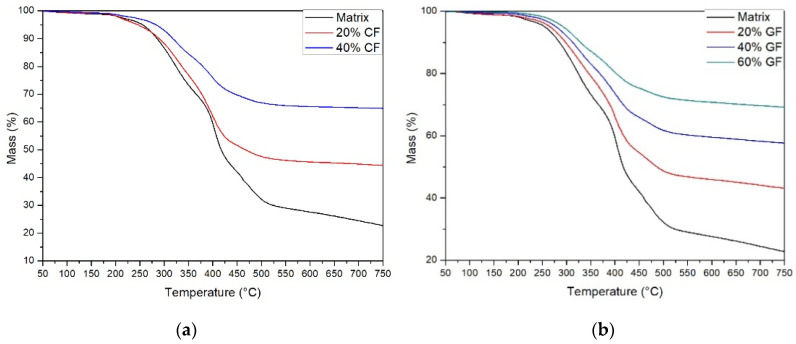
TGA curves of composites (**a**) carbon fiber, (**b**) glass fiber.

**Figure 5:**
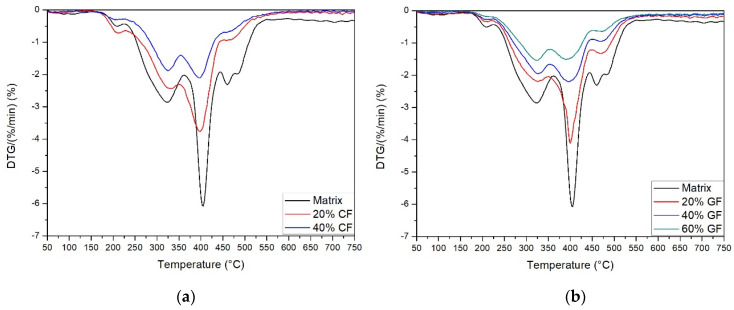
DTG (Derivative Thermogravimetry) curves of composites (**a**) carbon fiber, (**b**) glass fiber.

The thermogravimetric analysis curves of the obtained materials are presented in Figure 4 and Figure 5. Char residues, temperatures of two main steps of degradation, and temperatures of 2, 5, and 10% mass loss are presented in Table 5. The process of thermal decomposition of polyurethane materials is very complicated, due to the presence of a wide variety of substances, a large number of possible degradation products, especially for materials manufactured with the addition of bio-based polyols. Moreover, it is possible that the addition of bio-polyols in the production of PU materials leads to the deterioration of thermal stability [10,27].

In the polyurethane industry, it is generally accepted that a 2% mass loss in polyurethane is considered as the onset of the thermal degradation of materials. For samples with the addition of glass fiber, it was observed that the onset of the thermal degradation is significantly shifted to higher temperatures. It is also noticeable that the increasing amount of fiber also increases the onset of degradation. For the sample coded matrix, 40% GF and 60% GF onset temperatures were increased from 203.8 °C to 237.2 and 256.8 °C, respectively. For samples with a 40% addition of carbon fiber, T_2%_ increased to 222.5 °C. It can be concluded that the addition of inorganic fibers contributes to delaying the degradation of the material due to the higher thermal stability of fiber in comparison to the matrix, and restricting access to deeper layers of the material. The presence of inorganic fibers that degrade at high temperatures did not change the temperature of the main degradation steps. However, it resulted in a significant increase in the char residue after the test. This parameter was increased from 22.18% in the matrix up to 57.45 and 69.03% for 40% GF and 60% GF, respectively. The results are in line with other studies [28,29].

The collected data show that the obtained material degrades in a four-stage process. The peak of the first step of the degradation appears at the temperature of 200–220 °C and results from the unreacted polyol present in the structure of the material. The maximal temperature of the second step is between T_maxII_ = 320–330 °C and arises from the degradation of hard segments of polyurethane. During this step, the break of urethane bonds occurs and a number of products such as isocyanates, polyols, amines (primary and secondary), and carbon dioxide are generated [30,31].

The third peak with a maximal degradation rate at T_maxIII_ = 395–405 °C is associated with the degradation of segments formed by the bio-polyol such as poly (ethylene glycol) segments. Moreover, this step of degradation may be intensified by the decomposition of fatty acid chains in the bio-polyol structure [26,27,32]. The last stage of the degradation with a maximum at T_maxIV_ = 460–480 °C results from the thermolysis of organic products generated during previous degradation stages. Comparing the curves obtained for both types of composites, no significant differences during degradation were found and it was concluded that both fibers do not significantly influence the basic mechanism of the polyurethane degradation [28].

**Figure 6:**
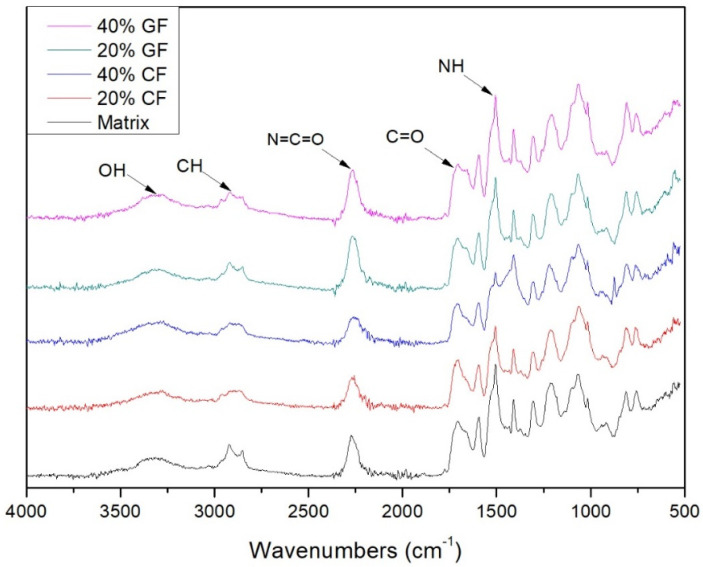
FTIR (Fourier transform infrared spectroscopy) spectra of the matrix.

To verify the structure of the bio-based matrix and composites, the FTIR (Fourier transform infrared spectroscopy) analysis was performed. Results are shown in Figure 6.

It can be observed that the spectra for matrix and glass/carbon fiber filled polyurethanes are very similar. For better visualization, only the matrix spectra will be described. A broad peak from around 3300 to 3400 cm^−1^ comes from OH stretching vibrations. Peaks around 2900 and 2800 cm^−1^ are from aliphatic CH_2_ symmetric and asymmetric stretching vibrations, respectively [30]. Stretching vibrations of carbonyl groups from urethane bond or carbonyl compounds from cellulose decomposition during the liquefaction process are present at around 1700 cm^−1^ [31]. From the received spectra, it can be concluded that the final product contained a significant amount of isocyanate groups, which is confirmed by the sharp and strong peak of the stretching vibrations of the isocyanate group at around 2250 cm^−1^. It can be related to the presence of NCO groups at the chain ends or with the presence of unreacted isocyanate [30]. However, the difference between glass and carbon fiber filled composites can be observed in this peak intensity. It can be observed that for glass fiber filled composites, the NCO peak is almost identical to the corresponding peak from the matrix. For carbon fiber filled composites, the NCO peak intensity decreases with the increasing amount of CF in the sample. It may be related to a much higher heat conductivity of carbon fibers compared to glass fibers and the matrix. All samples were thermally cured before the measurements. The higher heat conductivity of carbon fiber filled samples provided better heat distribution in the polymer matrix and improved the reaction speed of unreacted NCO groups [33]. The band at around 1500 cm^−1^ corresponds to the NH bending vibrations of the urethane group.

**Figure 7:**
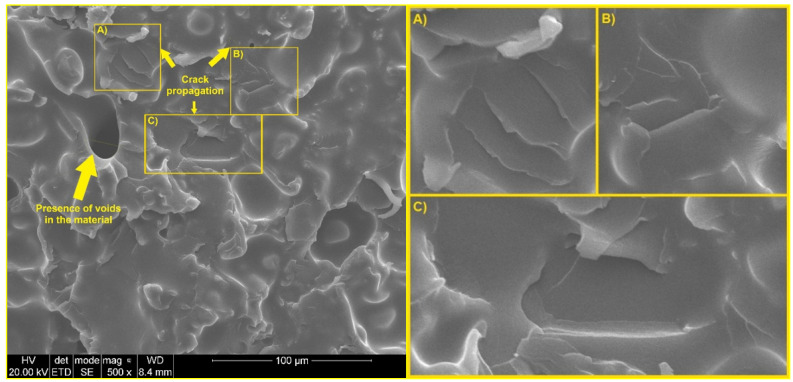
Scanning electron microscopy (SEM) images of the matrix.

**Figure 8:**
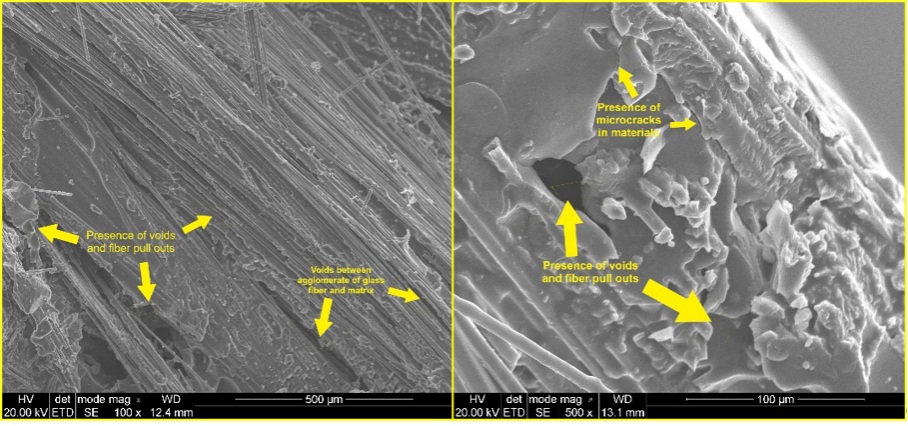
SEM images of the sample coded 50% GF.

**Figure 9:**
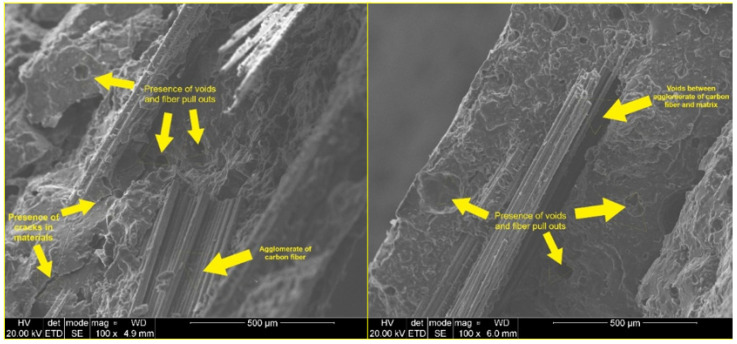
SEM images of the sample coded 50% CF.

Figure 7 shows the micrograph of the matrix, Figure 8 and Figure 9 show the micrographs of composites. 

**Scheme 1:**
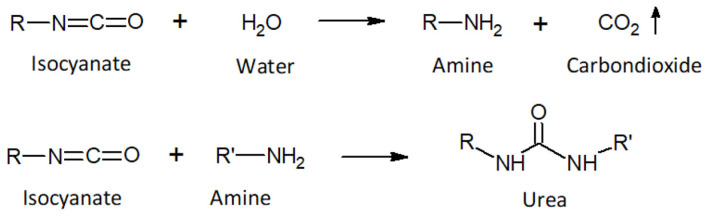
Reaction of uncontrolled foaming occurring during the manufacturing of materials.

During the analysis, a number of material defects can be noticed. One of the most important defects is the occurrence of voids and air bubbles in the material. Their presence is due to the incorporation of air during mixing of substances, unfilled gaps between the fibers (especially between carbon fibers due to electrostatic interactions), and the reaction of isocyanate with residual water which was not removed during the drying process. According to Scheme 1, during the reaction, CO_2_ is generated, which causes uncontrolled foaming of the material. Resulting voids can become centers of crack propagation in the material and reduce the cross-section and density of the tested material. Both effects can significantly affect the mechanical properties and impact the strength of composites.

The technological process of obtaining composites could also lead to the formation of glass/carbon fiber agglomerates in materials. The presence of agglomerates in the material may lead to the initiation of micro cracks and the further intensification of crack propagation through all directions in the composite. Moreover, it can cause non uniform stress transfer in the composite causing the stress concentration in the most sensitive areas of the composite. These phenomena lead to a faster brittle cracking of the material, significant reduction of the mechanical properties, and elasticity of the composite.

Moreover, the scanning electron microscopy (SEM) of the fractured samples shows the efficiency of wetting and the distribution of glass fibers. These parameters have a significant influence on the properties of composites containing fibers. If phases of the composite are well bonded, the applied stress could be properly transferred across the material including the matrix and fibers. The stress immediately propagates through the fiber and is then transferred back to the matrix. Poor fiber/matrix interfaces may result in the deterioration of material properties. The purpose of the addition of fibers is to enhance the stiffness and strength of the matrix. The main function of the matrix is to keep the fiber position and orientation in the composite, transmit shear force, protect the fiber surface, and transfer loads to the reinforcement. Summarizing, the functions of both phases are impossible to be fulfilled without proper adhesion [34]. In the case of inorganic fiber polyurethane composites, the nature of the adhesion between the polyurethane matrix and fiber is not clearly determined. It was shown that hydrogen bonding in an interphase region may have the greatest impact on the properties of the material such as flexural strength and impact strength. The same research showed that the interfacial interactions between the inorganic filler and polyurethane matrix are not so important. Therefore, the mechanical enhancement results from a good dispersion of fibers in the matrix and from an effective wetting [28].

The analysis of the images showed regions where glass and carbon fibers are not bonded to the matrix and the evidence of glass and carbon fiber pull-outs can be noticed. These phenomena may indicate that the adhesion of both fibers is not sufficient, which can be caused by the low surface area of the glass fiber (limited interaction area) and the absence of functional groups capable of reacting during the synthesis. Adhesion could be improved by the modification of the fiber surface or by the addition of a compatibilizer. As described above, this effect may affect the flexibility and mechanical properties of the obtained composites.

The analysis showed that the crack runs along the boundary of the fibers, which proves the limited adhesion between the fiber/matrix. Moreover, it may be due to the much higher strength of the fibers used than the matrix. Analyzing the structure of the matrix, unlike samples with the addition of fibers, one major crack propagation center cannot be found. A network of interconnecting microcracks can be seen, which may cause the fracture of the material.

## 4. Conclusions

In this work, the serviceability of cellulose derived bio-polyol obtained in the process of biomass liquefaction has been proven during the manufacturing of highly filled glass and carbon fiber composites. It has been successfully demonstrated that during a simple, nonmechanized method, it is possible to successfully obtain composites with useful properties, with a fiber content of up to 70%. This solution will be a greener alternative to the currently used composites produced only from petrochemical raw materials. This work also developed knowledge on interactions between the bio-based matrix and the filler, as well as the influence of inorganic fibers on the mechanical properties of these materials. Future studies should focus on the compatibilization of the proposed fibers, the addition of organic fibers into the matrix, and improvement of the manufacturing method. The conclusions are shown as follows:

The addition of fibers caused an increase in the mechanical properties such as stiffness, flexural strength, Young′s modulus, impact toughness, and an increase in hardness. On the other hand, the introduction of fibers to the polymer matrix caused the reduction of composites elasticity and decreased the maximal deformation at break.

Defects such as bubbles and voids, fiber pull-outs, fiber agglomerates, presence of cracks, and crack propagation lines were detected using scanning electron microscopy (SEM). These issues could be solved with an addition of anti-foaming agents and compatibilizer, mechanization of the mixing process or by more effective polyol dehydration.

The dynamic mechanical analysis (DMA) showed the increase in the storage modulus in a wide range of temperatures. Moreover, it was observed that the addition of fibers causes a shift in the glass transition temperature of the soft segments towards higher temperatures. This confirms the decrease in the polymer chain mobility.

The thermogravimetric analysis showed that the thermal stability of the composites was improved and this parameter increases with the greater addition of fiber. The temperatures of 2% mass loss were increased from 203.8 °C for the matrix up to 258.9 °C for 60% GF. The obtained material shows a four-step degradation with maximal degradation.

## Figures and Tables

**Table 1:** Composition of composites.

Sample	Content of Fiber	
	Glass Fiber	Carbon Fiber
Matrix	×	×
10% GF	10%	×
20% GF	20%	×
30% GF	30%	×
40% GF	40%	×
50% GF	50%	×
60% GF	60%	×
70% GF	70%	×
10% CF	×	10%
20% CF	×	20%
30% CF	×	30%
40% CF	×	40%
50% CF	×	50%

CF: Carbon fiber; GF: Glass fiber.

**Table 2:** Density, impact strength, and hardness of the obtained materials.

Sample	Density (g/cm)	Impact Strength (kJ/m^2^)	Hardness (Shore D)
Matrix	1.037 ± 0.010	6.01 ± 0.38	64.4 ± 1.0
10% GF	1.164 ± 0.009	8.13 ± 0.70	64.8 ± 1.0
20% GF	1.211 ± 0.010	9.19 ± 1.28	68.5 ± 1.3
30% GF	1.379 ± 0.035	13.62 ± 2.16	72.7 ± 2.2
40% GF	1.389 ± 0.009	19.96 ± 2.66	75.5 ± 0.8
50% GF	1.434 ± 0.014	27.32 ± 2.73	77.3 ± 1.3
60% GF	1.733 ± 0.051	25.40 ± 3.31	81.9 ± 1.6
70% GF	1.627 ± 0.020	15.19 ± 2.37	77.6 ± 1.1
10% CF	1.136 ± 0.017	8.96 ± 0.86	65.5 ± 2.2
20% CF	1.279 ± 0.014	13.49 ± 1.24	77.1 ± 1.7
30% CF	1.307 ± 0.026	16.00 ± 2.55	77.6 ± 1.9
40% CF	1.324 ± 0.016	16.53 ± 1.79	77.7 ± 2.1
50% CF	1.332 ± 0.025	22.78 ± 2.83	78.8 ± 3.2

**Table 3:** Flexural properties of the obtained materials.

Sample	Flexural Modulus (MPa)	Flexural Strength (MPa)	Maximal Deformation (%)
Matrix	450 ± 17	27.29 ± 1.54	9.87 ± 0.26
10% GF	620 ± 14	15.28 ± 0.88	4.86 ± 0.43
20% GF	976 ± 51	33.72 ± 2.91	4.71 ± 0.32
30% GF	1615 ± 35	47.35 ± 1.40	4.07 ± 0.58
40% GF	1560 ± 28	45.21 ± 0.13	4.60 ± 0.46
50% GF	2030 ± 84	58.15 ± 0.57	4.19 ± 0.30
60% GF	3885 ± 134	62.64 ± 1.54	2.54 ± 0.24
70% GF	2210 ± 129	32.69 ± 4.29	2.35 ± 0.37
10% CF	1426 ± 102	26.00 ± 1.18	2.20 ± 0.11
20% CF	3415 ± 148	71.14 ± 1.53	2.41 ± 0.18
30% CF	3895 ± 63	72.48 ± 1.53	2.66 ± 0.20
40% CF	3445 ± 49	45.31 ± 3.88	1.50 ± 0.19
50% CF	2495 ± 132	24.44 ± 0.80	1.37 ± 0.12

**Table 4:** Storage modulus at different temperatures and glass transition (Tg) of soft segments.

Sample	E’_20 °C_ (MPa)	E’_60 °C_ (MPa)	E’_100 °C_ (MPa)	1 Soft Segments T_g_ (°C)	2 Soft Segments T_g_ (°C)
Matrix	1493	550	375	−63.3	35.2
20% GF	2229	1047	638	−55.5	40.5
40% GF	3362	1872	1276	−52.2	45.3
60% GF	5667	2968	1873	−53.8	44.2
20% CF	3559	2064	1174	−51.1	42.5
40% CF	6756	4150	2526	−48.5	36.2

E’xx °C: Storage modulus at different temperatures; TGA: Thermogravimetric analysis.

**Table 5:** Results of thermogravimetric analysis of the obtained materials.

Sample	Different % Mass Loss in Temperature (°C)			T_maxII_ (°C)	T_maxIII_ (°C)	Char Residue after Test (%)
	T_2%_	T_5%_	T_10%_			
Matrix	203.8	255.4	285.3	321.4	403.0	22.18
20% GF	211.4	266.0	298.1	326.5	399.5	42.86
40% GF	237.2	278.7	312.7	325.7	394.9	57.45
60% GF	256.8	293.2	328.9	323.7	390.7	69.03
20% CF	202.1	245.2	290.2	329.8	398.0	44.27
40% CF	222.5	282.5	318.3	325.4	396.7	64.84

T’_maxII_: Temperature of maximal degradation at stage II; T’_maxIII_: Temperature of maximal degradation at stage III.

## Data Availability

Data available on request due to restrictions eg privacy or ethical. The data presented in this study are available on request from the corresponding author.
